# Supplementary material for: IL-6, NLR, and SII Markers and Their Relation with Alterations in CD8+ T-Lymphocyte Subpopulations in Patients Treated for Lung Adenocarcinoma
Source: Biology (Basel). 2020 Nov 5;9(11):376. doi: 10.3390/biology9110376 (PMC7694324; doi:10.3390/biology9110376)
Supplement: Supplementary file 1 [file biology-09-00376-s001.pdf]

Supplementary Table S1. Patients characteristics at 3rd and 6th cycle of treatment.

Characteristic	3rd cycle of treatment			*p*	6th cycle of treatment			*p*
	Total Group	Patients with:			Total Group	Patients with:		
		shorter survival[1]	longer survival[1]			shorter survival[1]	longer survival[1]	
n	53	22	31	-	53	22	31	-
**Clinical parameters**								
Leukocytes ($10^3/mm^3$)[2]	6.4 (5.3-8.2)	7.1 (5.4-10)	5.8 (4.9-8)	0.1346	6.2 (5.2-8)	7.8 (5.6-11.8)	5.8 (4.4-8.6)	0.0761
Lymphocytes ($10^3/mm^3$)[2]	1.8 (1.5-2.2)	1.7 (1.5-2.1)	1.8 (1.5-2.3)	0.9388	1.5 (1.1-2.3)	1.3 (0.9-2.3)	1.7 (1.4-2.2)	0.2094
TGF-β (ng/mL)[2]	19.5 (12.5-29.8)	16 (8.4)	19.6 (13.3-25.7)	0.2178	18.7 (12.2-27.8)	17.4 (13.3-27.2)	18.1 (10.8-27.6)	0.8074
HMGB-1 (ng/mL)[2]	1.9 (1.3-2.7)	1.9 (1.4-2.7)	1.9 (1.2-2.8)	0.8902	2 (1.4-2.9)	1.9 (0.9-2.7)	2 (1.4-3.0)	0.3895
IL-6 (pg/mL)[2]	3 (1.6-5.4)	3.7 (2.6-5.3)	2.7 (1.5-5.4)	0.3873	3.8 (1.9-8.1)	5.7 (2.2-12.9)	2.8 (1.8-6.4)	0.1701
NLR[2]	1.8 (1.5-2.5)	2.2 (1.6-3.5)	1.7 (1.5-2.2)	0.1274	2.1 (1.5-3)	2.9 (2-6.1)	1.8 (1.2-2.2)	0.0008
SII[2]	605.8 (432.8-813.2)	625.2 (433.7-818)	605.8 (414.5-834.2)	0.8346	593.4 (345.9-1093)	1062 (517.7-2278)	411.5 (293.6-729.5)	0.001
**T-lymphocytes (%)**[3] CD4+	39.45 ± 3.31	44.7 ± 6.49	36.82 ± 3.74	0.2746	41.81 ± 3.28	49.46 ± 4.75	37.56 ± 4.11	0.1599
Th1 (%)[3]	12.40 ± 2.57	15.38 ± 3.71	7.43 ± 2.31	0.0838	11.33 ± 2.34	13.61 ± 3.30	7.23 ± 2.41	0.1178
Th2 (%)[3]	2.81 ± 0.38	2.91 ± 0.49	2.63 ± 0.62	0.9070	2.77 ± 0.34	2.86 ± 0.41	2.62 ± 0.61	0.6888
Th17 (%)[3]	3.42 ± 0.39	3.18 ± 054	3.88 ± 0.51	0.2198	3.71 ± 0.47	3.39 ± 0.52	4.28 ± 0.96	0.4081
Treg (%)[3]	4.43 ± 0.46	4.71 ± 0.59	3.91 ± 0.74	0.5251	4.10 ± 0.35	4.12 ± 0.41	4.08 ± 0.68	0.8322
CD8+ (%)[3]	26.87 ± 2.48	17.60 ± 1.95	31.50 ± 3.06	0.0533	25.47 ± 1.86	19.06 ± 1.48	29.03 ± 2.40	0.0025
CTLs GrB+Perf+ (%)[3]	37.54 ± 3.91	25.29 ± 3.76	43.66 ± 5.01	0.072	38.39 ± 3.98	2625.34 ± 6.49	45.64 ± 4.	0.0085

[1] Patients were categorized according to median OS. [2] Median (interquartile range). [3] Mean ± SEM. NLR = Neutrophil-lymphocyte ratio. SII = Systemic immune-inflammatory index. GrB = Granzyme B. Perf = Perforin.

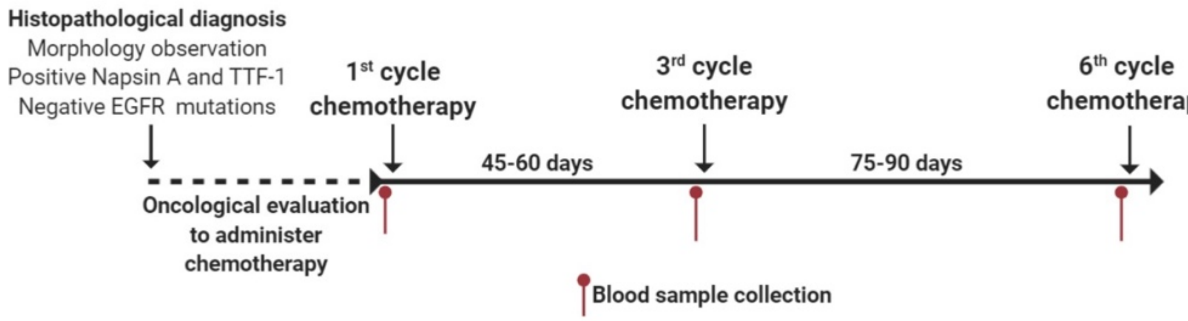

Supplementary Figure S1. Schematic representation of lung adenocarcinoma patient's treatment. The first, third, and sixth cycle of treatment is indicated. Also, the blood sample collection is shown. Created with BioRender.com.

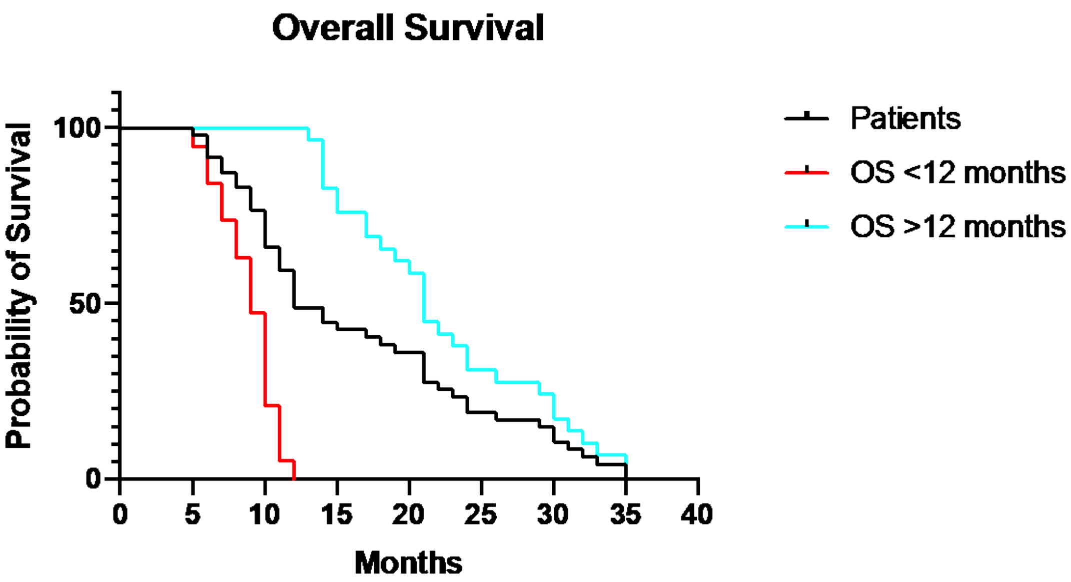

Supplementary Figure S2. Kaplan-Meier overall survival (OS) analysis. Total patients (*n* = 53), patients with shorter (< 12 months, *n* = 22) and longer (> 12 months, *n* = 31) OS are shown.

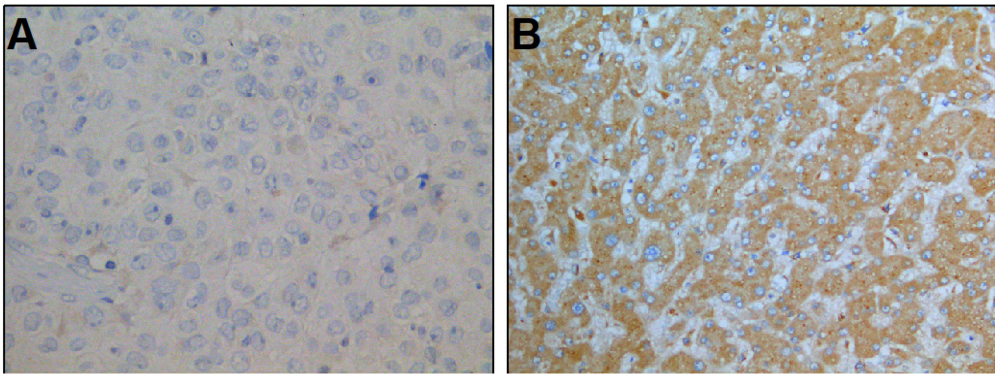

Supplementary Figure S3. Negative and positive controls for IL-6 staining. (A) Tumor tissue without primary antibody (Negative control) and (B) IL-6 staining in liver biopsy (Positive control) are shown. Magnification 200X.

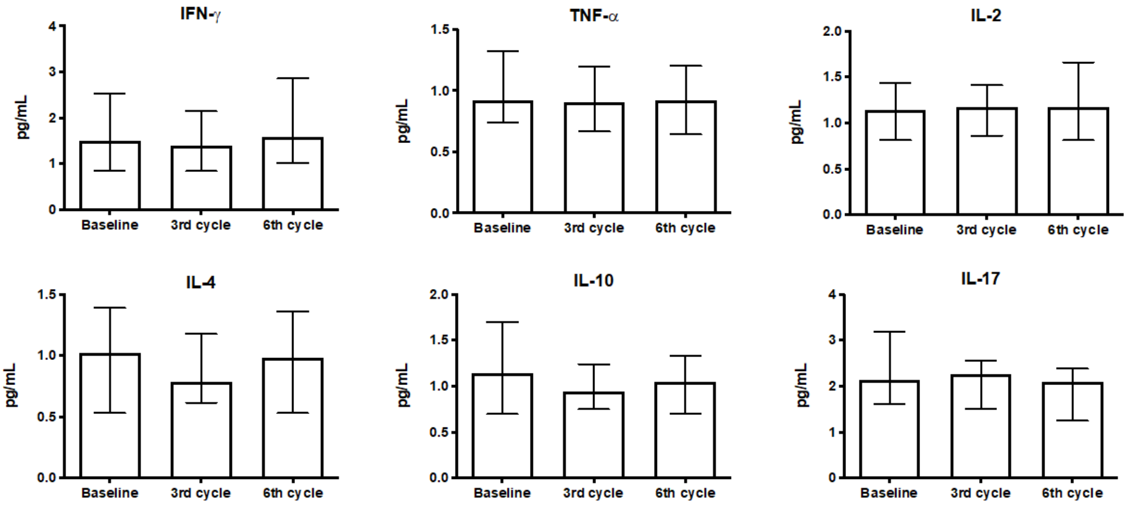

Supplementary Figure S4. IFN-g, TNF-a, IL-2, IL-4, IL-10 and IL-17 plasma concentrations in patients throughout treatment cycles. *n* = 53. Median and interquartile ranges are shown.
